# Plastid caseinolytic protease OsClpR1 regulates chloroplast development and chloroplast RNA editing in rice

**DOI:** 10.1186/s12284-021-00489-6

**Published:** 2021-05-20

**Authors:** Xi Liu, Ziyi Xu, Yanrong Yang, Penghui Cao, Hang Cheng, Haiying Zhou

**Affiliations:** 1 Key Laboratory of Eco-Agricultural Biotechnology around Hongze Lake, Regional Cooperative Innovation Center for Modern Agriculture and Environmental Protection, Huaiyin Normal University, Huai’an, 223300 China; 2 Suzhou Academy of Agricultural Sciences, Suzhou, 215155 China

**Keywords:** *Oryza sativa*, OsClpR1, Chloroplast development, Chloroplast RNA editing

## Abstract

**Background:**

Plant plastidic caseinolytic protease (Clp) is a central part of the plastid protease network and consists of multiple subunits. The molecular functions of many Clps in plants, especially in crops, are not well known.

**Results:**

In this study, we identified an albino lethal mutant *al3* in rice, which produces albino leaves and dies at the seedling stage. Molecular cloning revealed that *AL3* encodes a plastid caseinolytic protease, OsClpR1, homologous to *Arabidopsis* ClpR1 and is targeted to the chloroplast. Compared with the wild type, chloroplast structure in the *al3* mutant was poorly developed. *OsClpR1* was constitutively expressed in all rice tissues, especially in young leaves. The *OsClpR1* mutation affected the transcript levels of chlorophyll biosynthesis and chloroplast development-related genes. The RNA editing efficiency of three chloroplast genes (*rpl2*, *ndhB*, *ndhA*) was remarkably reduced in *al3*. Using a yeast two-hybrid screen, we found that OsClpR1 interacted with OsClpP4, OsClpP5, OsClpP2, and OsClpS1.

**Conclusions:**

Collectively, our results provide novel insights into the function of Clps in rice.

**Supplementary Information:**

The online version contains supplementary material available at 10.1186/s12284-021-00489-6.

## Background

Chloroplasts are a semi-autonomous organelle responsible for photosynthesis, and the biosynthesis and storage of multiple metabolites (Moreira et al. 2000; Sugimoto et al. 2004). The functional chloroplast is derived from the proplastid, and is synergistically regulated by the plastid and nuclear genomes (López-Juez 2007; Sakamoto et al. 2008). Accumulating evidence indicates that protein degradation plays an important role in chloroplast biogenesis (Clarke et al. 2005; Kato and Sakamoto 2010).

Plastids contain several proteases, such as the stromal Ser Clps, the thylakoid-bound FtsH metalloproteases, and EGY1 proteases (Adam et al. 2006; Zheng et al. 2006). The ATP-dependent Clp peptidase has been studied, and the plastid Clp proteolytic system in plants consists of five ClpP proteins, four ClpR proteins, and three Clp chaperones (ClpC1, ClpC2, and ClpD; Constan et al. 2004; Sakamoto 2006; Sjögren and Clarke 2011). Additionally, plants have two specific accessory ClpTs and an adaptor ClpS (Sjögren and Clarke 2011). Recent studies have reported that plant chloroplast Clps are involved in controlling chloroplast development and plant growth. In *Arabidopsis thaliana*, the loss of function mutants *clpp4* and *clpp5* are embryonic lethal, while the loss of function mutant *clpp3* is seedling lethal (Kovacheva et al. 2007; Kim et al. 2013). The *ClpC1* knockout mutant exhibits growth retardation and leaf chlorosis, while there was no obvious phenotype in the *clpC2* mutant (Zhang et al. 2018). However, the *clpC1*/*clpC2* double mutant exhibits defects in embryogenesis. In rice, the leaves of the homozygous mutant *osclpP5* were light yellow, and died at the three-leaf stage (Tsugane et al. 2006). A rice yellow leaf mutant *vyl* showed a yellowing phenotype during the whole growth period, and then gradually turned green from the top to the bottom (Dong et al. 2013). *VYL* encodes a subunit of the plastid caseinolytic protease homologous to the *Arabidopsis* ClpP6 subunit. In addition, the *OsClpP6* mutation affected plant height, panicle length, and leaf morphology (Li et al. 2013). However, little is known about the structure and function of chloroplast Clps in rice.

In this study, we isolated a rice albino lethal mutant *al3* with decreased chlorophyll contents and impaired chloroplasts. *AL3* encodes a subunit of the plastid caseinolytic protease, OsClpR1, and influenced the transcription of chlorophyll biosynthesis and chloroplast development-related genes. Notably, OsClpR1 affected the chloroplast RNA editing of *rpl2*, *ndhB*, and *ndhA*. Furthermore, using the yeast two-hybrid analysis, we found that OsClpR1 interacted with OsClpP4, OsClpP5, OsClpP2, and OsClpS1.

## Results

### Characterization of the *al3* Mutant in Rice

**Fig. 1:**
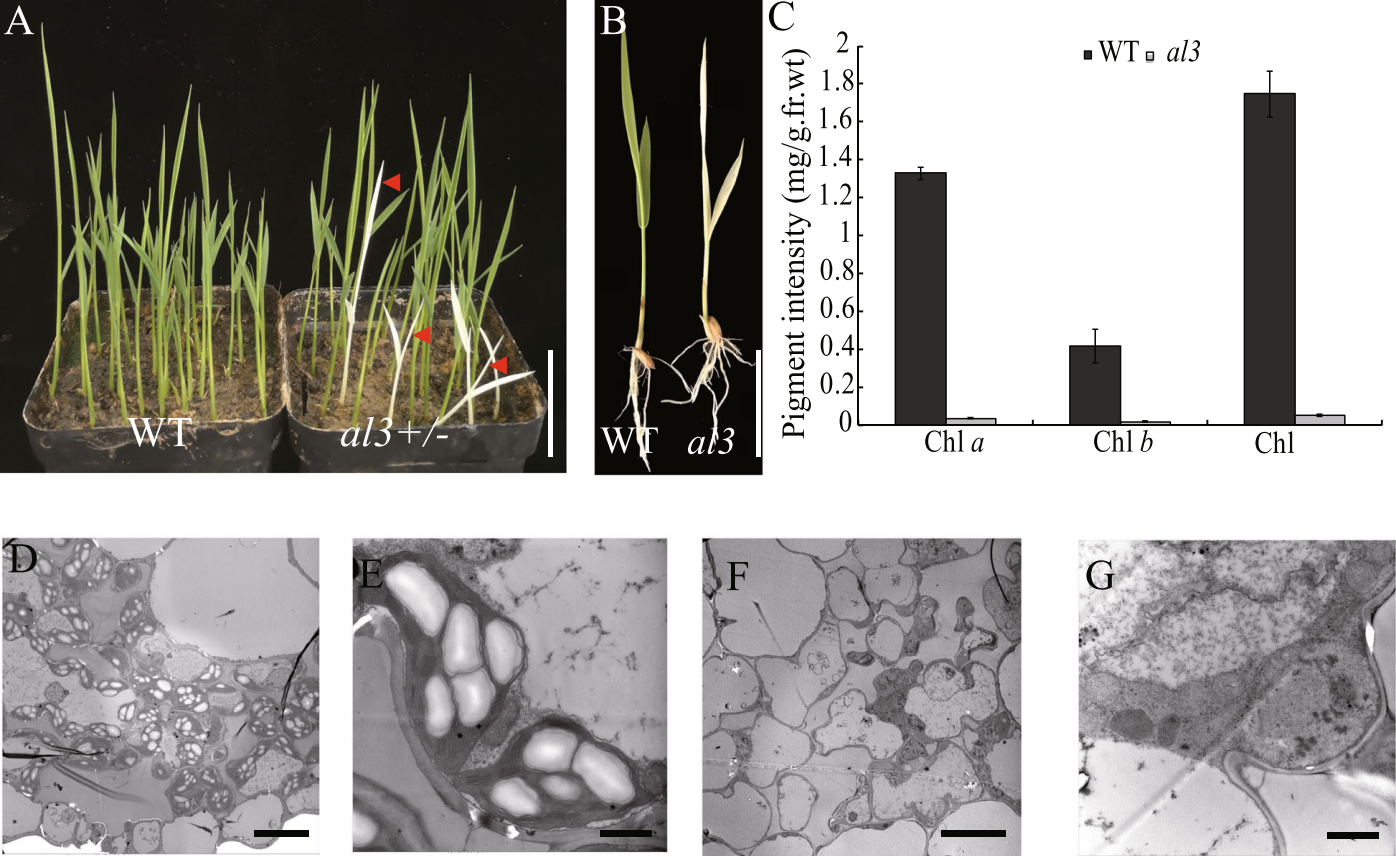
Phenotypes of the wild-type and the *al3* mutant. **a**, **b** Leaf color of the 7-day-old wild-type and *al3* seedlings. Bars = 5 cm. **c** Chlorophyll contents of 7-day-old wild-type and *al3* seedlings. **d**, **e** Chloroplast ultrastructure in the wild type mesophyll cell. **f**, **g** Chloroplast ultrastructure in *al3* mesophyll cell. Bars = 10 μm for (**d**) and (**f**); 1 μm for (**e**) and (**g**)

We isolated a leaf color mutant *albino lethal 3* (*al3)* by screening the T-DNA insertion library in rice (Dongjin background). The *al3* mutant exhibited the albino phenotype and could not be recovered at a later developmental stage compared with wild-type (Fig. 1a, b). Eventually, the *al3* plants died. Consistent with the albino leaf phenotype of *al3*, the chlorophyll *a* and *b* contents were reduced significantly in *al3* (Fig. 1c).

To further investigate the leaf color phenotype between wild-type and *al3*, we performed TEM to observe and compare the chloroplast ultrastructure of the *al3* mutant and wild-type. The chloroplasts of *al3* leaves were poorly developed and lacked organized thylakoids (Fig. 1d, e), whereas the development of chloroplasts in wild-type leaves were well developed and had well-structured thylakoids (Fig. 1f, g). Taken together, the *AL3* gene is essential for rice chloroplast development.

### Molecular Cloning of the *AL3* Gene

**Fig. 2:**
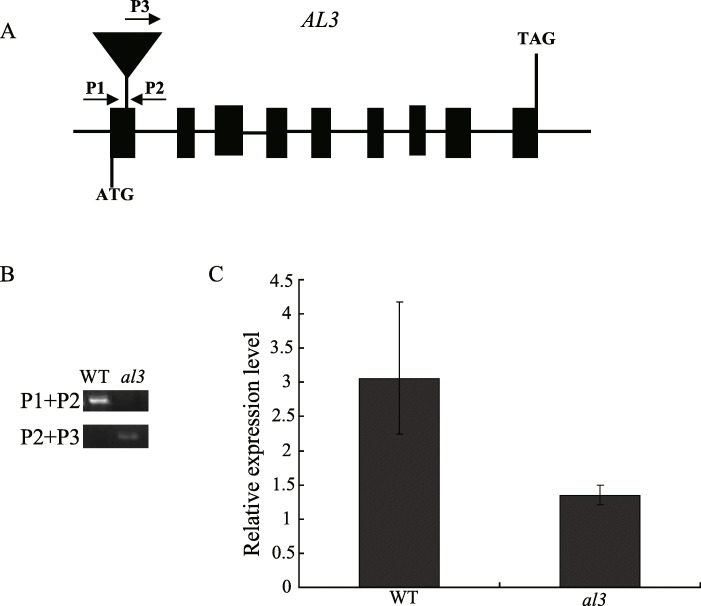
Molecular cloning of *AL3*. **a** Schematic representation of the *AL3* gene. Black boxes represent exons, and the lines represent introns. T-DNA is inserted into the first exon of *LOC_Os05g51450*. **b** PCR verification of the T-DNA insertion site. **c** Expression level of *AL3* in the wild-type and *al3*

**Fig. 3:**
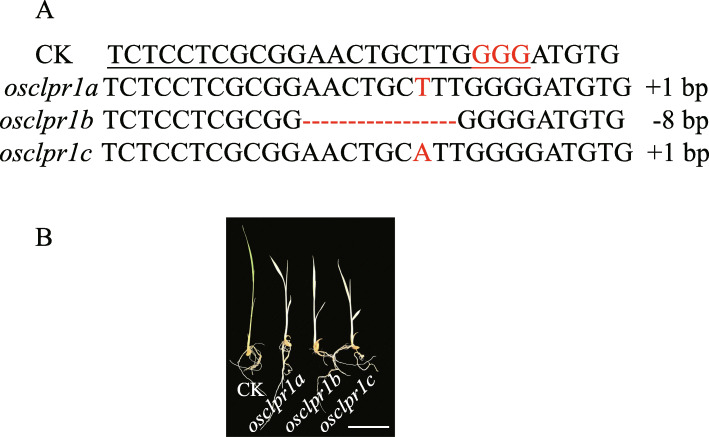
CRISPR/Cas9-targeted mutagenesis of *AL3*. **a** Mutation of the knockout lines. The 20-bp gene-specific target sequences and PAM are underlined and in red. The number of nucleotides deleted and inserted are indicated by the minus (−) and plus (+) signs, respectively. **b** Comparisons of leaf color in the knockout lines. CK indicates transgenic negative plants. Bars = 5 cm

To clone the gene for *al3*, we used tail-PCR to obtain the flanking sequence in *al3*. Only one fragment sequence was aligned with the rice gene *LOC_Os05g51450*, suggesting that one T-DNA was located in *al3*. Furthermore, we identified a genomic flanking sequence by searching the T-DNA insertion database (http://orygenesdb.cirad.fr/). Subsequently, we designed three PCR primers (P1, P2, and P3) and identified the T-DNA insertion site in *al3* (Fig. 2a, b). The result suggested that the T-DNA was located in the first exon of *LOC_Os05g51450* (Fig. 2a, b), which consists of nine exons and eight introns (Fig. 2a). To explore the influence of the T-DNA insertion on *LOC_Os05g51450*, we analyzed the expression level of *LOC_Os05g51450*. The transcript level of *LOC_Os05g51450* was significantly reduced by the T-DNA insertion in *al3* (Fig. 2c). Therefore, we speculated that *LOC_Os05g51450* was the candidate *AL3* gene. To confirm that *LOC_Os05g51450* was the candidate *AL3* gene, we used CRISPR/Cas9 to knock out *LOC_Os05g51450* in the Nipponbare background. We sequenced *LOC_Os05g51450* in the 25 T_0_ transgenic plants and obtained six heterozygous mutants which were similar to the leaf color of wild-type plants. Then, three T_1_ transgenic homozygous plants were obtained, which exhibited albino leaves (Fig. 3a, b). In addition, we detected the potential off-target sites and did not find any mutations in any of the potential off-target sites (Additional file 1: Table S1). These results revealed that *LOC_Os05g51450* is the *AL3* gene.

### AL3 Is a Plastid-Localized and Conserved Clp Protease

**Fig. 4:**
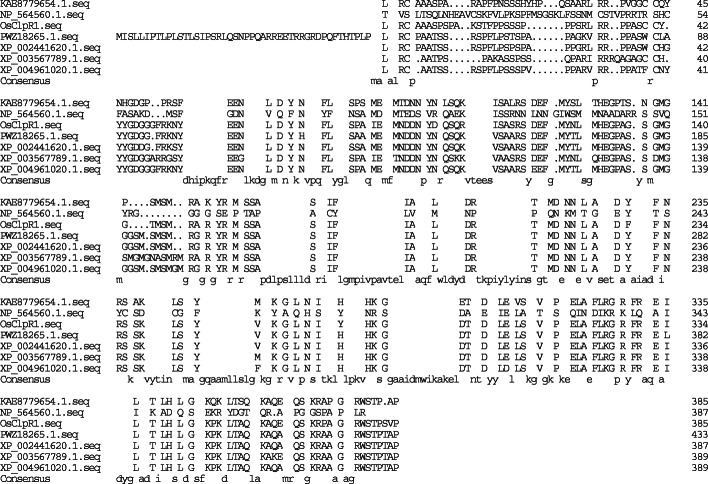
Amino acid sequence alignment of OsClpR1 and its homologs PWZ18265.1 (*Zea mays*), XP_002441620.1 (*Sorghum bicolor*), XP_004961020.1 (*Setaria italica*), XP_003567789.1 (*Brachypodium distachyon*), KAE8779654.1 (*Hordeum vulgare*), NP_564560.1 (*Arabidopsis thaliana*). Amino acids that are fully or partially conserved are shaded black and pink, respectively

Sequence and bioinformatic analysis indicated that the *AL3* contains nine exons and eight introns (Fig. 2a). *AL3* is predicted to encode a Clp protease of 386 amino acids. A BLAST search of the sequence suggested that the protein encoded by *AL3* shares 52.5% amino acid sequence identity with the plastid ClpPR protease ClpR1 in *Arabidopsis thaliana* (Kim et al. 2009; Fig. 4). Therefore, we named AL3 as OsClpR1. The *ClpR1* mutant developed slower than the wild type with pale green leaves and affected chloroplast development (Koussevitzky et al. 2007). Amino acid comparison indicated that proteins homologous to OsClpR1 are highly conserved among *Zea mays*, *Hordeum vulgare*, *Sorghum bicolor*, *Setaria italica*, *Brachypodium distachyon* (Fig. 4).

**Fig. 5:**
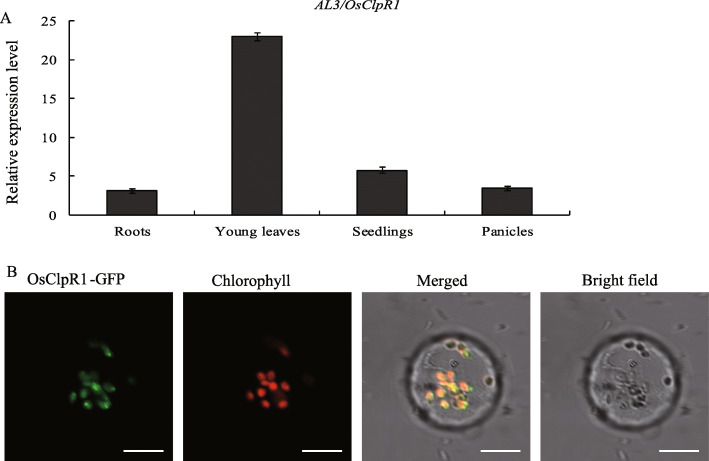
Expression and subcellular localization of OsClpR1. **a** Expression of *OsClpR1* in roots, young leaves, panicles, and 7-day-old seedlings of wild-type plants. **b** Subcellular localization of OsClpR1. Green, red and yellow fluorescence show GFP, chloroplast autofluorescence, and the merged fluorescence, respectively

ChloroP analysis revealed that OsClpR1 has a chloroplast transit peptide at the N-terminus with 40 amino acids (www.cbs.dtu.dk/services/ChloroP/). To verify this prediction, we constructed two transformation vectors expressing the OsClpR1-GFP and OsClpR1^41–386^-GFP fusion protein and introduced them into rice protoplasts. The results showed that the green fluorescence of OsClpR1-GFP fully co-localized with the Chl fluorescence of the chloroplasts (Fig. 5b). However, OsClpR1^41–386^-GFP fluorescence was observed in the cytosol (Additional file 1: Figure S2). These results indicated that the N-terminal 40 amino acids are essential for OsClpR1 to target the chloroplasts.

### Expression Pattern of *AL3*

To analyze the expression pattern of *AL3*, we first searched the *AL3* gene in the Rice eFP Browser (http://bar.utoronto.ca/efprice/cgi-bin/efpWeb.cgi), and found that *OsClpR1* was expressed in various tissues, and especially in leaves (Additional file 1: Figure S2). To test the result of the prediction, we carried out qRT-PCR to analyze the *OsClpR1* expression in roots, young leaves, seedlings, and panicles. The result indicated that *OsClpR1* was constitutively expressed in various tissues (Fig. 5a).

### Altered Expression of Chlorophyll Biosynthesis and Chloroplast-Associated Genes in *al3*

We observed that chloroplasts were impaired in *al3* and hypothesized that the loss of function of OsClpR1 may influence the expression of chlorophyll biosynthesis and chloroplast-associated genes. To test this hypothesis, we carried out qRT-PCR to analyze the expression of these genes. Our results indicated that the expression levels of three tetrapyrrole biosynthesis genes (*HEMA*, *HEMC*, and *HEME*), three subunits of Mg-chelatase (*CHLH*, *CHLI*, and *CHLM*), magnesium-protoporphyrin IX monomethyl ester cyclase *CRD*, and divinyl reductase *DVR* were remarkably reduced in *al3*, compared with wild-type (Additional file 1: Figure S3A). In addition, the expression levels of chloroplast-associated genes, e.g. *psaA*, *atpB*, and *rps2*, were significantly down-regulated (Additional file 1: Figure S3B). Taken together, our results revealed that the OsClpR1 may coordinate the chlorophyll biosynthesis and chloroplast-associated genes to regulate rice chloroplast development.

### Altered RNA Editing of *rpl2*–1, *ndhA*-1019, *ndhB*-611 and *ndhB*-737 in *al3*

**Fig. 6:**
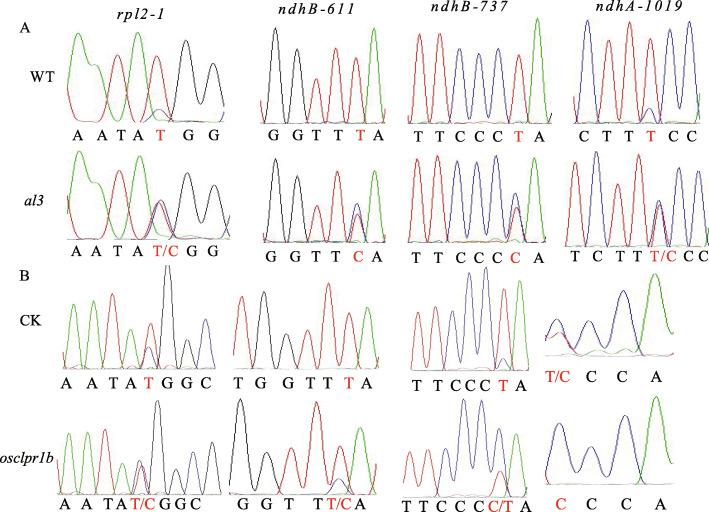
OsClpR1 is required for chloroplast RNA editing at four sites. **a** Chloroplast RNA editing analysis of the wild type and *al3*. **b** RNA editing analysis of *rpl2*, *ndhB* and *ndhA* in the *OsClpR1* knockout lines

Previous studies have shown that plastid RNA editing plays an important role in regulating chloroplast development in plants (Xiao et al. 2018; Huang et al. 2020; Wang et al. 2020). To verify whether the *OsClpR1* mutation affects chloroplast RNA editing, we examined the RNA editing efficiency of 21 editing sites in wild-type and *al3*. We observed that the RNA editing of *rpl2*–1, *ndhA*-1019, *ndhB*-611 and *ndhB*-737 in *al3* were significantly reduced compared to wild-type (Fig. 6a), whereas the other 17 editing sites were not affected (Additional file 1: Figure S4).

In addition, we examined the editing efficiency of *rpl2*–1, *ndhA*-1019, *ndhB*-611 and *ndhB*-737 in the CRISPR/Cas9 knock-out lines. RNA editing at these four editing sites was also reduced in the CRISPR/Cas9 knock-out lines (Fig. 6b). These results suggest that the *OsClpR1* mutation affects chloroplast RNA editing in rice.

### Interactions between OsClpR1, OsClpP2, OsClpP4, OsClpP5, and OsClpS1

**Fig. 7:**
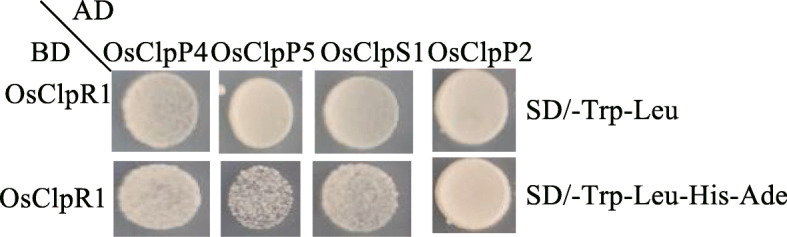
Interaction between OsClpR1 and OsClpP4, OsClpP5, OsClpP2, and OsClpS1 by the yeast two-hybrid analysis. *OsClpR1* was fused to the pGBKT7 vector (OsClpR1-BD). *OsClpP4*, *OsClpP5*, *OsClpP2*, and *OsClpS1* were fused to the pGADT7 vector

To investigate the role of OsClpR1 in the assembly of the rice Clp complex assembly, we carried out a yeast two-hybrid screen to identify the proteins that directly interact with OsClpR1. We cloned the rice homologs of the *Arabidopsis* protease complex and named them as OsClpR2 (Os06g0136800), OsClp5(Os03g0344900), OsClpS1(Os08g0432500), OsClpP2(Os04g0525600), OsClpP3(Os01g0507900), OsClpP4(Os10g0580800), OsClpP5(Os03g0308100), and OsClp-T (Os03g0247000). As shown in Fig. 7, OsClpR1 interacted with OsClpS1, OsClpP2, OsClpP4, and OsClpP5, but not with OsClpR2, OsClp5, OsClpP3, VYL and OsClp-T (Additional file 1: Figure S5A-B). This result indicated that OsClpR1 likely serves as a core component in the assembly of the rice Clp complex. OsClpP4 and OsClpT are reported to interact with themselves and form homodimers (Dong et al. 2013). Therefore, we used yeast two hybrid analysis to verify whether OsClpR1 can form a homodimer. However, OsClpR1 could not interact with itself to homodimerize (Additional file 1: Figure S5C).

## Discussion

Plastid caseinolytic protease has been extensively studied in dicotyledonous plants, but it has been scarcely studied in monocotyledonous plants, especially in rice (Zhang et al. 2018). In this study, we isolated and identified a rice plastid caseinolytic protease protein OsClpR1. The *OsClpR1* gene was highly expressed in young leaves, and lowly expressed in roots and panicles. Knockout of *OsClpR1* caused a significant decrease of chlorophyll contents and functional chloroplasts. OsClpR1 was located in chloroplasts and is conserved among *Zea mays*, *Hordeum vulgare*, *Sorghum bicolor*, and *Arabidopsis thaliana*. In summary, our results provide novel insights for the regulation of chloroplast development by Clps in rice. Leaf color is one of the important factors affecting photosynthesis, which directly affects rice yield. To date, more than 120 genes have been reported to regulate chloroplast development in rice, such as *YGL8*, *TSC1*, and *Lhca4* (Kong et al. 2016; Shi et al. 2018; Yamatani et al. 2018). The expression levels of chlorophyll biosynthesis and plastid-encoded genes in the *al3* mutant were remarkably reduced compared with the wild type (Additional file 1: Figure S3). Our study was helpful to improve the molecular mechanism of Clps regulating chloroplast development in rice.

In addition, we observed defects in RNA editing of *rpl2*–1, *ndhA*-1019, *ndhB*-611 and *ndhB*-737 in the *al3* mutant (Fig. 7a), whereas all other chloroplast editing sites were similar to the wild-type (Additional file 1: Figure S3). Additionally, the RNA editing of *rpl2*–1, *ndhA*-1019, *ndhB*-611 and *ndhB*-737 in CRISPR/Cas9 knockout plants were also reduced (Fig. 7b). RNA editing, as a posttranscriptional modification, is essential for generating mature transcripts of plastid genes (Yan et al. 2018). However, not all albino mutants in rice exhibit chloroplast RNA editing defects in rice. For example, the rice albino mutant *wsl3* has almost no impairment in chloroplast RNA editing (Wang et al. 2020). Previous studies have shown that multiple organellar RNA editing factor (MORF) proteins are essential for plastid and mitochondrion RNA editing in *Arabidopsis* and rice (Bentolila et al. 2012; Zhang et al. 2017). The MORF protein WSP1 is involved in the editing of six plastid RNA editing sites in rice (Zhang et al. 2017). To investigate whether OsClpR1 affects RNA editing by interacting with MORFs, we used the yeast two-hybrid screen to examine the interaction between OsClpR1 and the 7 MORF proteins in the rice genome. Unfortunately, OsClpR1 did not interact with any of the seven MORF proteins in the screen (data not shown), suggesting that OsClpR1 influences chloroplast RNA editing independent of an interaction with MORFs. Our future studies will be focus on identifying the interacting proteins of OsClpR1 to elucidate the mechanism of its regulation of chloroplast RNA editing.

## Conclusions

OsClpR1 regulates chloroplast development in rice and influences chloroplast RNA editing. Our findings contribute to understanding the mechanisms of rice development and the functional characterization of Clps in monocots.

## Methods

### Plant Materials

The *al3* mutant (1A-08939) was isolated from a T-DNA insertion library with the cv Dongjin background (Jeon et al. 2000; Jeong et al. 2006). The mutant and wild-type plants were planted in paddy fields under natural conditions in Huaian, China.

### Chl Content Determination and Transmission Electron Microsocpy (TEM)

Leaves from wild-type and *al3* plants were collected at the seedling stage. Approximately 200 mg of powdered leaves were soaked in 20 mL of 95% ethanol for 48 h in the dark. Chl contents for each sample were measured in triplicate as previously described (Liu et al. 2020).

For TEM, leaves from wild-type and *al3* plants were collected and fixed in 2.5% (v/v) glutaraldehyde. The sample preparation was performed as previously described (Liu et al. 2020). The chloroplast structure of leaf cells was observed under a JEOL 1200EX transmission electron microscope.

### Identification of T-DNA Insertion Locus and CRISPR/Cas9 Knock-out of *AL3*

To identify the T-DNA insertion locus in *al3*, we searched the flanking sequence database (Jeong et al. 2006; http://orygenesdb.cirad.fr/). The T-DNA locus was detected by PCR with the primers P1, P2, and P3 (Additional file 1: Table S2).

To knock out *AL3*, one CRISPR/Cas9 vector was constructed as previously described (Lu et al. 2017). The recombinant vector was transformed into rice cv Nipponbare by *Agrobacterium*. The genotype of CRISPR/Cas9 plants was analyzed by PCR and the amplification products were sequence-verified. Genome target sequences and PCR primers are listed in Additional file 1: Table S2.

### RNA Extraction and Quantitative RT-PCR

Total RNA from roots, young leaves, seedlings at the two-leaf stage, and panicles were isolated by the RNAprep Pure Plant Kit (CWBIO, Jiangsu, China). First-strand cDNA was generated from 1 μg of total RNA using the PrimeScript 1st Strand cDNA Synthesis Kit (TaKaRa). Quantitative RT-PCR was performed as previously described and the primers used to measure the expression level of chlorophyll biosynthesis and chloroplast-associated genes were obtained from the study by Liu et al. 2020. The primers used for the expression level of *OsClpR1* are listed in Additional file 1: Table S2.

### Subcellular Localization of OsClpR1

To confirm the subcellular localization of OsClpR1, the coding region of *OsClpR1* (without the stop codon) was introduced into the pAN580-GFP vector and transformed into rice protoplasts. To detect the chloroplast signal of the OsClpR1 protein, and one truncated OsClpR1 segment were inserted into the pAN580-GFP vector, termed OsClpR1^41–386^-GFP. Fluorescence was observed by a Zeiss LSM700 confocal laser-scanning microscope. The primers used for constructing the vector are listed in Additional file 1: Table S2.

### Analysis of RNA Editing

Specific cDNA fragments containing editing sites were amplified and sequenced. The cDNA sequences were compared to identify C to T changes resulting from RNA editing as previously described (Tan et al. 2014; Cui et al. 2019; Huang et al. 2020).

### Yeast Two-Hybrid Analysis

The full-length cDNA of *OsClpR1* and nine Clp genes were cloned into pGBKT7 and pGADT7, respectively. The vectors were transformed into yeast strain AH109 according to the manufacturer’s instructions (Clontech). The primers used for constructing the vectors are listed in Additional file 1: Table S2.

## Supplementary Information

**Additional file 1: Figure S1.** Subcellular localization of OsClpR1^41-386^-GFP. **Figure S2.** Expression pattern of *OsClpR1* at various growth periods. Data was collected from the Rice eFP Browser. **Figure S3.** Expression analysis of chlorophyll biosynthesis and chloroplast development related-genes in wild-type and *al3*. **Figure S4.** The rest 17 RNA editing sites in wild-type and *al3*. **Figure S5.** A yeast two-hybrid interaction assay between OsClpR1 and five Clp proteins in rice. **Table S1.** Off-target effect detection. **Table S2.** Primers used in this study.

## Data Availability

All data supporting the conclusions of this article are provided within the article (and its additional files).

## Abbreviations

Chl: Chlorophyll
Chl *a*: Chlorophyll *a*
Chl *b*: Chlorophyll *b*
qRT-PCR: Quantitative real-time polymerase chain reaction
MORF: Multiple organellar RNA editing factor
Clp: Plastidic caseinolytic protease
TEM: Transmission electron microsocpy
WT: Wild type
