# Supplementary material for: Plastid caseinolytic protease OsClpR1 regulates chloroplast development and chloroplast RNA editing in rice
Source: Rice (N Y). 2021 May 20;14:45. doi: 10.1186/s12284-021-00489-6 (PMC8137786; doi:10.1186/s12284-021-00489-6)
Supplement: Supplementary file 1 — Additional file 1: Figure S1. Subcellular localization of OsClpR141-386-GFP. Figure S2. Expression pattern of OsClpR1 at various growth periods. Data was collected from the Rice eFP Browser. Figure S3. Expression analysis of chlorophyll biosynthesis and chloroplast development related-genes in wild-type and al3. Figure S4. The rest 17 RNA editing sites in wild-type and al3. Figure S5. A yeast two-hybrid interaction assay between OsClpR1 and five Clp proteins in rice. Table S1. Off-target effect detection. Table S2. Primers used in this study. [file 12284_2021_489_MOESM1_ESM.doc]

###### Supplementary

###### Supplemental Tables S1 and five figures

**RUNNING TITLE:** *OsClpR1* regulates chloroplast development in rice

Plastid caseinolytic protease OsClpR1 regulates chloroplast development and influence chloroplast RNA editing in rice

**Xi Liu^1*^, Ziyi Xu^1^,Yanrong Yang^1^, Penghui Cao^2^ , Hang Cheng^1^, Haiying Zhou^1^**

*^1^Key Laboratory of Eco-Agricultural Biotechnology around Hongze Lake, Regional Cooperative Innovation Center for Modern Agriculture and Environmental Protection, Huaiyin Normal University, Huai’an 223300, China.*

*^2^Suzhou Academy of Agricultural Sciences, Suzhou 215155, China.*

Corresponding author:

Xi Liu

E-mail: 1240623244@qq.com

**
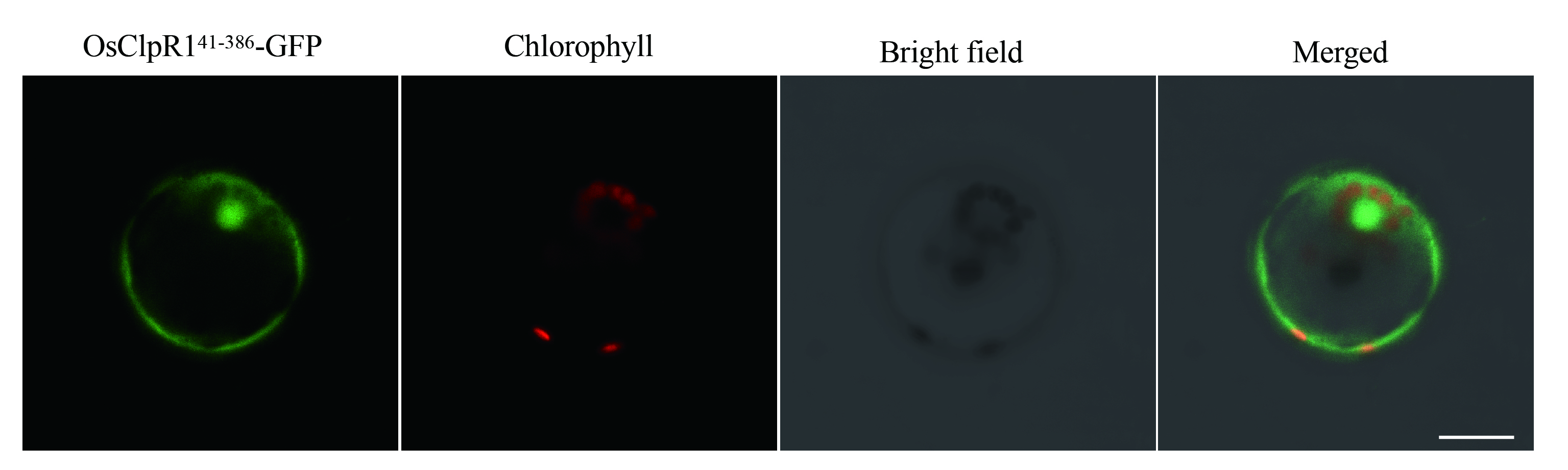
**

Supplemental Figure 1. Subcellular localization of OsClpR1^41-386^-GFP.

**
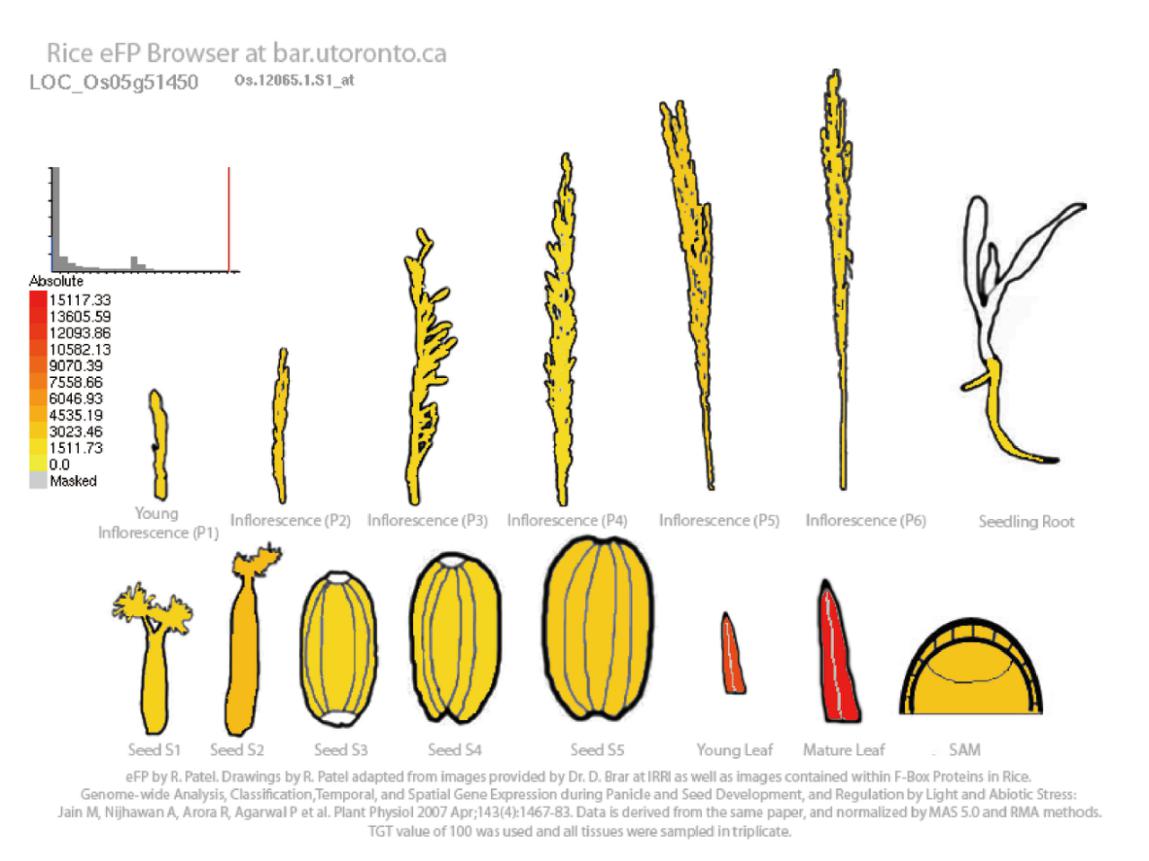
**

Supplemental Figure 2. Expression pattern of *OsClpR1* at various growth periods. Data was collected from the Rice eFP Browser.


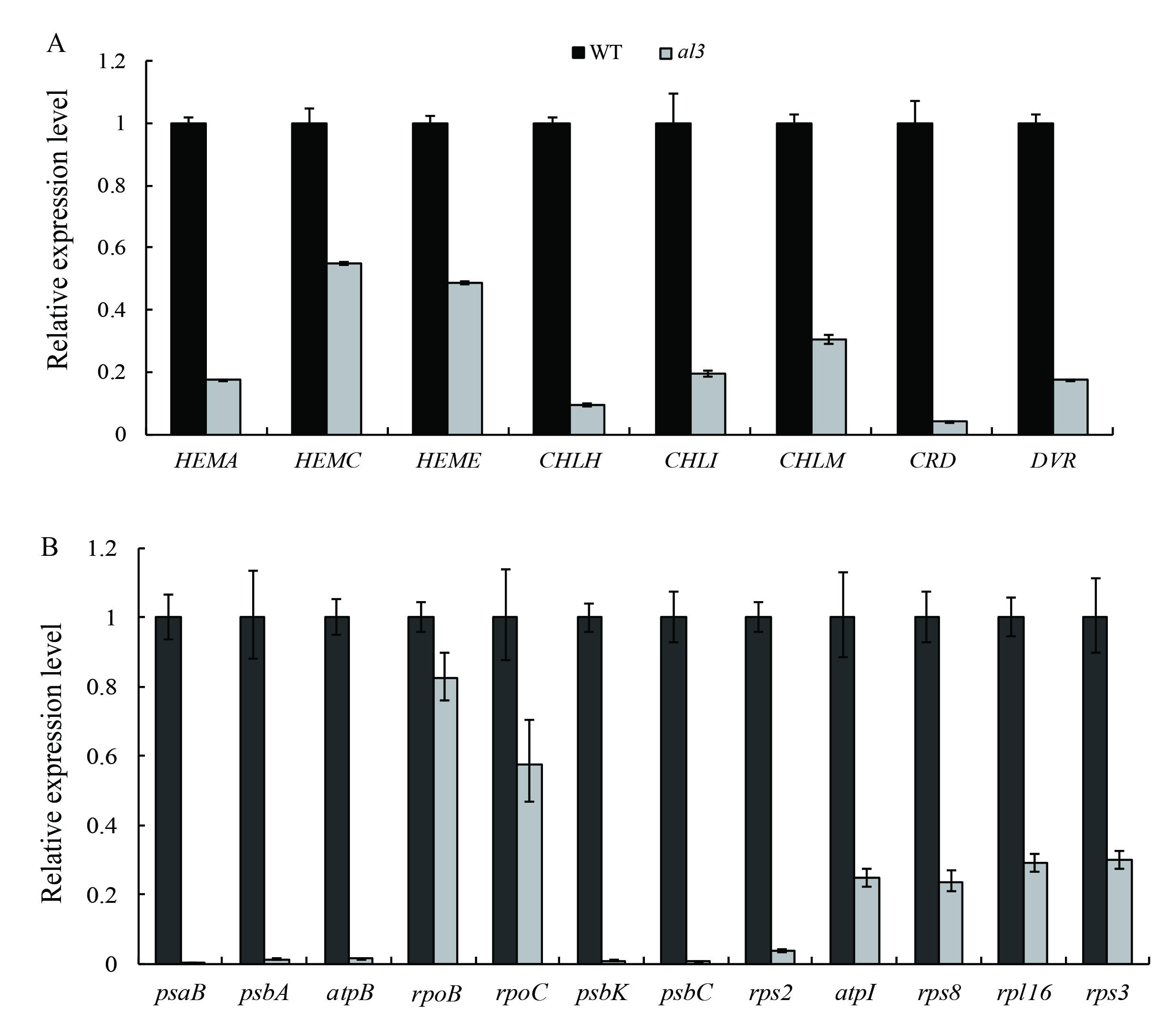


Supplemental Figure 3. Expression analysis of chlorophyll biosynthesis and chloroplast development related-genes in wild-type and *al3*.


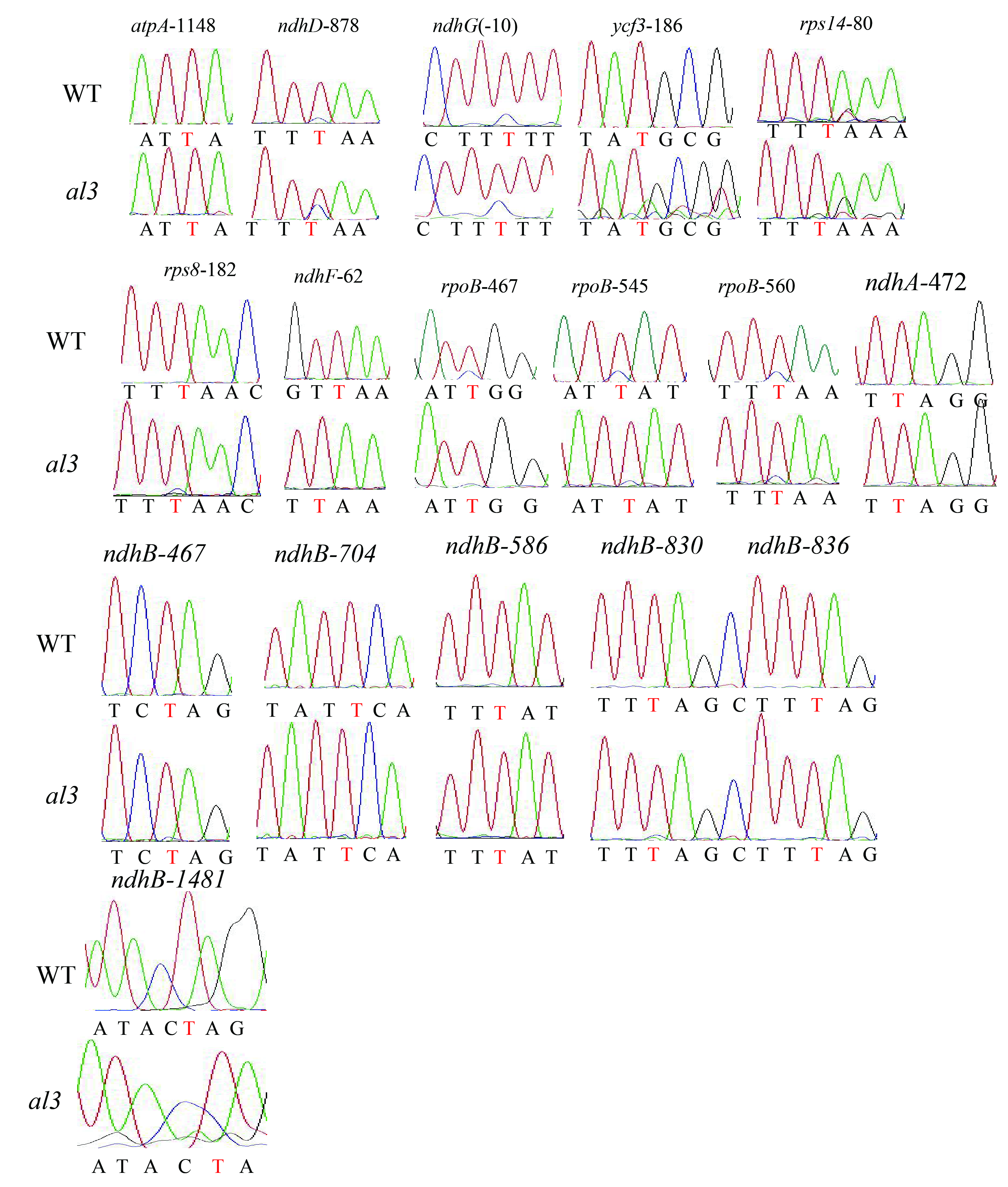


Supplemental Figure 4. The rest 17 RNA editing sites in wild-type and *al3*.

**
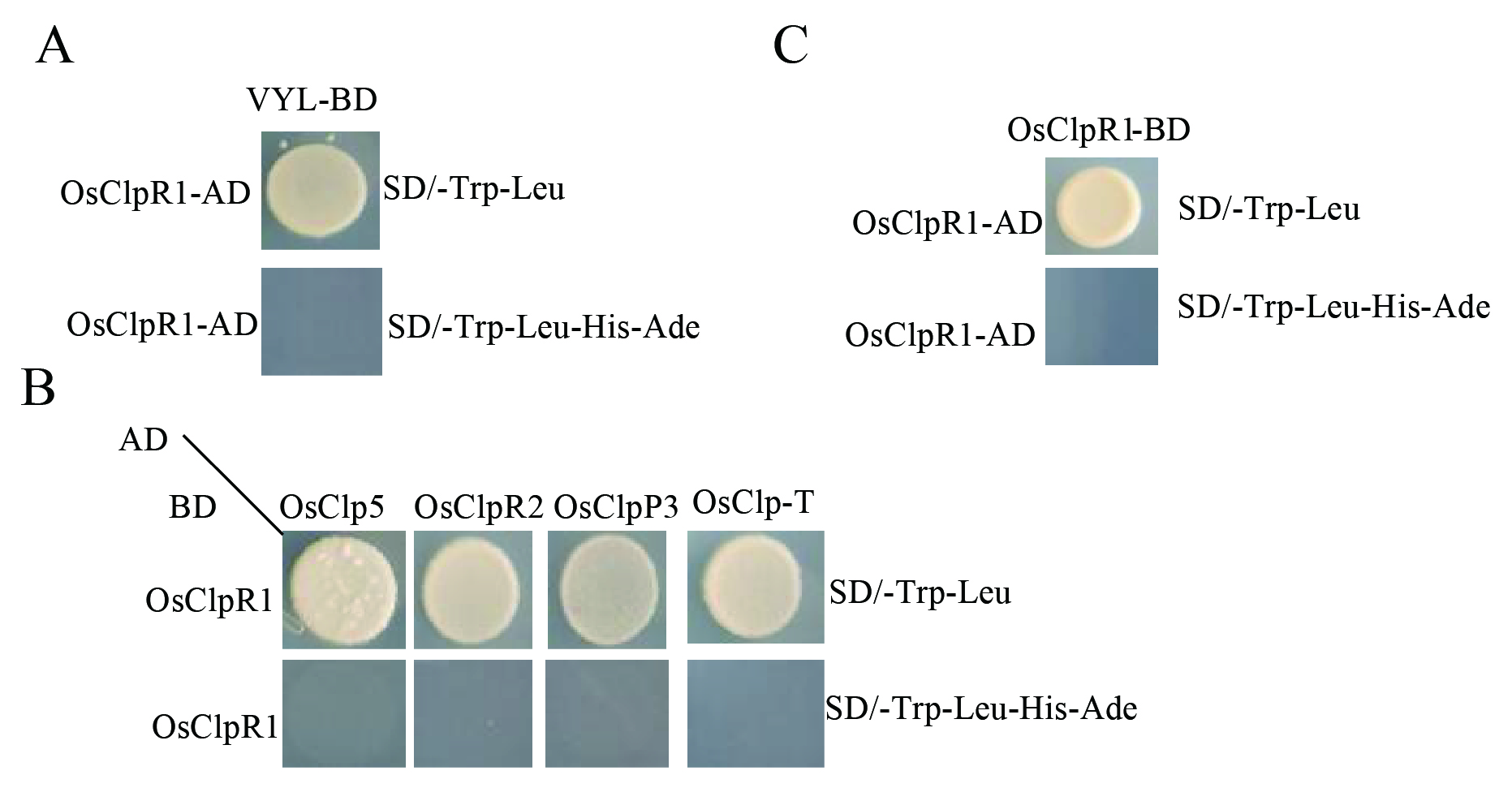
**

Supplemental Figure 5. A yeast two-hybrid interaction assay between OsClpR1 and five Clp proteins in rice. (A) Interaction assay between OsClpR1 and VYL.(B) Interaction assay between OsClpR1 and OsClp5, OsClpR2, OsClpP3, and OsClp-T. (C) OsClpR1 did not form homodimer.

Supplemental Table 1 Off-target effect detection

| **Sequence** | **MMs** | **Gene** | **Region** | **Muations** |
| --- | --- | --- | --- | --- |
| TCTC**G**TCGCGG**C**ACTGCTT**C** | 3 | Os03g0570300 | exon | No |
| TC**G**C**G**TCGCGGAACT**C**CTTG | 3 | Os06g0691500 | cds | No |
| TCTCCTC**T**C**T**GA**GA**TGCTTG | 4 | Os01g0857700 | exon | No |
| **G**CTCCTCG**A**GGAACTGCT**CC** | 4 | Os08g0243600 | exon | No |
| **C**CTC**T**TCGCGGA**C**CT**C**CTTG | 4 | Os11g0135400 | exon | No |

Supplemental Table 2 Primers used in this study

| Marker | Forward primer sequence (５′ →３′ ) | Revers primer sequence （５′ →３′ ） |
| --- | --- | --- |
| AL3-P1 | TATCCAAATCCCAGTGAAGCGAAGG | / |
| AL3-P2 | CCAAGCCATGTGGCGAAACCT | / |
| AL3-P3 | AACGCTGATCAATTCCACAG | / |
| OsClpR1-Crispr | GGCACAAGCAGTTCCGCGAGGAGA | AAACTCTCCTCGCGGAACTGCTTG |
| OsClpR1g | AAGCCATGTGGCGAAACCTA | CGAAGGCCACCAATCCTCTT |
| OsClpR1-BD-F | CATGGAGGCCGAATTCATGGCGCTCGCGCTGCGCTG | GCAGGTCGACGGATCCCTATCTAGGGACACTTGGAGT |
| OsClpP3-AD-F | TGCGGGATCCATGGAGGCAGCGGCAGCCATG | GCTGAGCTCAGCTGTTGAGATCTCCTTGAACTTC |
| OsClpR1-AD-F | GGAGGCCAGTGAATTCATGGCGCTCGCGCTGCGCTG | CGAGCTCGATGGATCCCTATCTAGGGACACTTGGAGT |
| VYL-BD | CATGGAGGCCGAATTCATGGCGCCTATGGCCATCTC | GCAGGTCGACGGATCCTTAGTATCTTGTTTCCAGCAG |
| OsClpP4-AD | GGAGGCCAGTGAATTCATGTCGGCTGCGAGTGCGAG | CGAGCTCGATGGATCCCTAGTATATCTCATCATCTG |
| ClpS1-AD | GGAGGCCAGTGAATTCATGGAGGCGGCGGTGCCCAG | CGAGCTCGATGGATCCTCAGCAGCCACCACTCGCAG |
| ClpR2-AD | GGAGGCCAGTGAATTCATGGCGCTGTCCGCGGCGGC | CGAGCTCGATGGATCCCTAGCCAAGTCCCAGATTAC |
| ClpP2-AD | GGAGGCCAGTGAATTCATGCTCCCCGTGGCCCCCAC | CGAGCTCGATGGATCCTCAAGCTGGAGATGGTTCAT |
| VYL-AD | GGAGGCCAGTGAATTCATGGCGCCTATGGCCATCTC | CGAGCTCGATGGATCCTTAGTATCTTGTTTCCAGCAG |
| OsClp5-AD | GGAGGCCAGTGAATTCATGGCGTCCGCCTCCCTGTC | CGAGCTCGATGGATCCTCACATAATATCAGGGCGTCT |
| OsClpT-AD | GGAGGCCAGTGAATTCATGGCGACCGCCGCCCAGGC | CGAGCTCGATGGATCCCTATCTAGGACTCATAGCAG |
| OsClpP5-AD | GGAGGCCAGTGAATTCATGGCGACCACCACCACCACC | CGAGCTCGATGGATCCCTAGAAGCAGGAAGCGGTTGAAG |
| OsClpR1-pAN580 | CGGAGCTAGCTCTAGAATGGCGCTCGCGCTGCGCTG | TGCTCACCATGGATCCTCTAGGGACACTTGGAGTTGA |
| OsClpR1^41-386^-pAN580 | CGGAGCTAGCTCTAGAATGTGCTACTACTACGGCGACGG | TGCTCACCATGGATCCTCTAGGGACACTTGGAGTTG |
| OsClpR1-qRT | TCAGGCTGCAATGCTTCTGTC | TCAAGGTAGTAATCCGTGTTCGTG |
